# Presence of a dominant native shrub is associated with minor shifts in the function and composition of grassland communities in a northern savannah

**DOI:** 10.1093/aobpla/plab011

**Published:** 2021-02-23

**Authors:** Isaac Peetoom Heida, Charlotte Brown, Margarete A Dettlaff, Kenneth J Oppon, James F Cahill

**Affiliations:** Department of Biological Sciences, CW 405 Biological Sciences Building, University of Alberta, Edmonton, AB, T6G 2E9, Canada

**Keywords:** architecture, composition, grassland, parkland, savannah, shrub, structure, understory

## Abstract

Ecosystems are spatially heterogenous in plant community composition and function. Shrub occurrence in grasslands is a visually striking example of this, and much research has been conducted to understand the functional implications of this pattern. Within savannah ecosystems, the presence of tree and shrub overstories can have significant impacts on the understory herbaceous community. The exact outcomes, however, are likely a function of the spatial arrangement and traits of the overstory species. Here we test whether there are functional linkages between the spatial patterning of a native shrub and the standing biomass, community composition, and overall nutrient cycling of a neighbouring grassland understory communities within the Aspen Parkland of central Alberta, Canada. In a paired grassland-shrub stand study, we found the native shrub, *Elaeagnus commutata*, has relatively few stand-level impacts on the composition and standing biomass of the ecosystem. One factor contributing to these limited effects may be the overdispersion of shrub stems at fine spatial scales, preventing areas of deep shade. When we looked across a shrub density gradient and incorporated shrub architecture into our analyses, we found these shrub traits had significant associations with species abundance and root biomass in the understory community. These results suggest that stem dispersion patterns, as well as local stand architecture, are influential in determining how shrubs may affect their herbaceous plant understory. Thus, it is important to incorporate shrub spatial and architectural traits when assessing shrub-understory interactions.

## Teaser

Shrubs can have differential impacts on neighboring plants. Species diversity and ecosystem can have a large influence on the exact effects a shrub may have on its surroundings, making comparisons between studies difficult. Here, we test whether shrub spatial arrangement and architecture is associated with impacts on the surrounding plant community. Compared to binary shrub-grassland analyses, our trait-based approach was able to detect significant associations between shrub presence and plant community function. Our shrub architecture approach is likely applicable to other shrub-focussed research and may help to increase our understanding of patterns of shrub presence in a variety of ecosystems.

## Introduction

Many ecosystems contain heterogenous subcomponents, each with distinct plant communities and functions. In savannah type ecosystems, the presence or absence of tall woody species can play a dramatic role in the structure and function of low-lying herbaceous plant communities. Understanding how shrubs influence plant communities has gained increased interest over the past decades, in part due to the dramatic negative effects often associated with increases in shrub density (Van Auken 2009). Reports of negative shrub impacts have traditionally arisen from ecosystems where there is little historic woody cover (Eldridge *et al.* 2011). In these scenarios, shrubs present a novel architecture and growth form that can alter the functioning of the ecosystem (Knapp *et al.* 2008), an outcome often viewed as negative in economically important grasslands. However, even without encroachment, understanding the structure and function of shrub dominated communities is important as these communities can have distinct ecological functionality within the broader landscape. In areas where shrublands, grasslands and forests all coexist, shrublands can act as intermediate successional stages in transitions between grasslands and forests (Archer *et al.* 1988; Aerts *et al.* 2006). The complexity and heterogeneity of these grassland–shrubland–forest ecotones also results in temporal instability, where small changes to disturbance or climatic regimes can result in dramatic shifts in the relative abundance of each component of the system (Camill *et al.* 2003). In these systems, it is important to understand the function of each component type, so that we can better understand the implications of dramatic community-cover shifts on landscape level functionality.

Shrublands can develop from grasslands in response to shifts in the disturbance regime such as reductions in fire frequency and overgrazing (Van Auken 2009). Anthropogenic activity has perpetuated both of these changes, increasing the likelihood for grasslands to change into shrublands. As shrubs expand into new areas, they can alter soil nutrient cycles (Jackson *et al.* 2002; Baer *et al.* 2006; Zhou *et al.* 2019), change light and moisture availability (Scott *et al.* 2006; Brantley and Young 2007), influence plant community composition (Báez and Collins 2008), and alter the physical characteristics (such as topography) of the habitat (Parizek *et al.* 2002). Shrubs can also interact with trees, and there is evidence both of facilitation of tree establishment (Smit *et al.* 2008) as well as suppression of tree growth (Berkowitz *et al.* 1995). The exact outcomes of shrub presence in a given system are hard to predict (Eldridge *et al.* 2011) as they are contingent on multiple local abiotic and biotic factors such as precipitation (Knapp *et al.* 2008), grazing pressure (Eldridge and Soliveres 2015) and the traits of the shrub itself (Eldridge *et al.* 2011). Shrub traits likely have consequences for plant community assembly as they determine the exact effects of the shrub on resource availability, creating assembly filters for understory communities. Shrub canopies overtop low-lying grassland vegetation, intercepting light and reducing light availability below the canopy (Brantley and Young 2007), sometimes favouring the establishment of shade-tolerant species (Yang *et al.* 2010). Shrubs’ height and rigid stems also allow for the creation of refugia under their canopies, as they can ameliorate stressors common in grassland systems. Shrubs can reduce drought severity (Cruz-Alonso *et al.* 2020) and abate the negative effects of livestock grazing (Bailey 1970; Eldridge *et al.* 2015; Perea *et al.* 2017) as well as acting as nurse plants after fire events (Raffaele and Veblen 1998). The reductions in disturbance severity under shrub canopies, coupled with light reductions, might interact to create an assembly filter selecting for shade-tolerant species, resulting in a compositional shift away from a natural grassland and towards either a shrub-dominated state (Lett and Knapp 2003) or composition more typical of a forested system (Dalotto *et al.* 2018). However, the degree to which shrubs act as assembly filters through resource modification should be a direct consequence of the architecture and spatial organization of the establishing shrub.

Savannah ecosystems represent a unique context for quantifying differences between shrubbed and shrubless areas. Defined by the presence of scattered woody vegetation in a grassland matrix, savannahs are held in (dis)equilibrium by resource limitation and disturbance regimes (Accatino *et al.* 2010). The permanent presence of woody species in savannahs contrasts with grassland systems which typically have much lower woody cover (in the absence of encroachment) (Van Auken 2000). The Aspen Parkland region of North America, characterized by patches of aspen forest (*Populus tremuloides* Michx.) interspersed within a grassland matrix, provides a good system to examine shrub-grassland associations because it is a transitionary zone between the great plains to the south and boreal forest to the north (Bird 1930). This system naturally experiences transitions between woody and non-woody community types (Pinno and Wilson 2011), with the rate of transition being influenced by the effects of grazing (Bork *et al.* 2013), drought (Hogg *et al.* 2008) and fire (Bailey *et al.* 1990). Changes in these disturbance regimes due to climate change (Schneider *et al.* 2009) or human activity (Michaelian *et al.* 2011) will likely lead to changes in the relative abundance of woody plants within the savannah matrix.

Of the woody parkland species, the shrub wolf-willow (*Elaeagnus commutata* Bernh. ex Rydb.) has a set of traits that will likely make its impacts on understory plant community assembly unique. Wolf-willow is an actinorhizal nitrogen-fixing shrub (Visser *et al.* 1991), reproducing clonally via its root system to form continuous stands rather than occurring as a single or a few isolated stems. Increased fertility via nitrogen fixation could interact with shrub canopy light interception to modify community assembly filters within the local grassland (Stevens *et al.* 2017). Wolf-willow is also an ‘increaser’ in overgrazed rangelands (USDA 2020) with density typically controlled by fire and moderate grazing/mowing (Arnold and Higgins 1986). Combined anthropogenic fire suppression and changes to grazing regimes have the potential to drive changes in the abundance of this shrub. At least one study has reported increases in abundance of wolf-willow in fescue grasslands (Bailey 1970); however, the potential impacts on assembly filters and plant understory community composition remain unclear. Information on how wolf-willow communities differ from adjacent shrubless grasslands is sparse and more data is needed to understand the pattern and processes associated with wolf-willow presence in grasslands. In this study, we aim to test the impacts of wolf-willow on plant community composition, understory biomass, and nutrient cycling within a native grassland. We build on previous work that has documented grassland community shifts in response to wolf-willow (Whysong and Bailey 1975) by incorporating the architecture of wolf-willow stands and examining its effects on both biotic and abiotic properties of the grassland. Here we answer three main questions:

1) Do wolf-willow and grassland plots differ in core aspects of biomass, fertility and biodiversity?
2) Are aspects of wolf-willow architecture associated with observed variation in understory biomass, fertility and biodiversity?
3) Does wolf-willow show evidence of acting as an assembly filter?

## Methods

### Study site

The study occurred in a 50-hectare field at the Roy Berg Kinsella Research Ranch in Alberta, Canada (53.0848°, -111.5600°). The site is in the Aspen Parkland ecoregion and the dominant grassland species include *Festuca hallii*, *Hesperostipa curtesita* and *Poa pratensis* (Lamb 2008). The site has historically been lightly grazed by cattle, with the last grazing event occurring 3 years prior to this study. Within the site, wolf-willow stands occur both at forest margins as well as in core grassland communities.

### Study design

**Figure 1.**
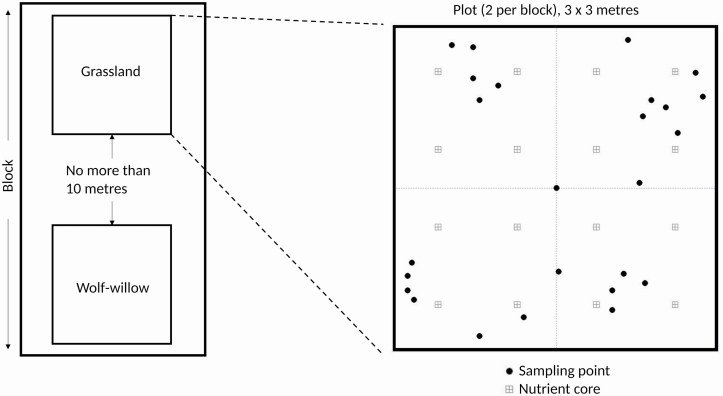
Sampling design schematic for examining patterns of wolf-willow presence in the Aspen Parkland. On the left, a conceptual diagram of how plots were arranged within blocks. Community type designation is written in each plot. On the right, an expanded view of the sampling design within one plot. Solid circles are the points at which images were taken and biomass and soil moisture measured. Hollow squares designate the location of soil cores for nutrient analysis.

We established 10 replicate blocks that were each comprised of two 3 × 3 m plots. These plots sizes were chosen to optimize our ability to detect local community differences; however, we recognize they may reduce our ability to measure landscape-level patterns. These paired plots were separated by <10 m, with one located in a wolf-willow stand and the second in a nearby native grassland with <1 % wolf-willow cover. We avoided selecting paired plot locations that bordered aspen stands to avoid potential confounding effects from aspen stands. Blocks were separated by at least 30 m, with plots within blocks placed randomly within the suitable grassland and wolf-willow regions. Within each block, we had three scales of measurement: the two plots, quadrants within plots and points within plots. Quadrants were created by subdividing each plot into four subplots, each 1.5 × 1.5 m. Point measurements were taken at a series of 25 10 cm diameter points laid out identically within each plot (Fig. 1). This pattern is subset of the sampling design used in Chagnon *et al.* (2018). We generated a series of random subsets of their sampling point pattern, and selected a pattern based on the pair-wise distance distribution and the number of points per quadrant. We selected for the pattern that had both a relatively flat distribution (increasing replication at nearest and furthest pair-wise distances) and relative parity in the number of points per quadrant. Table 1 displays the final structure of our study, outlining which data were measured at each scale.

**Table 1.** Scales of measurement in this study, and the corresponding measurements taken at each scale.

Scale	Data
Plot	Wolf-willow map
	Wolf-willow architecture
	• Height
	• Basal area
	• Canopy area
Quadrant	Soil nitrogen
	Soil moisture
	Soil pH
Point	Above-ground biomass
	Below-ground biomass
	Litter mass
	Soil moisture
	Canopy pictures (Wolf-willow only)
	Community composition (Presence/Absence)

### Wolf-willow structure and spatial characteristics

To characterize the architecture of wolf-willow stands, all wolf-willow stems in plots were mapped to the nearest centimetre. Additionally, we recorded basal area, maximum stem height and canopy area for each stem. Canopy area was calculated as the oval area of the widest diameter and the corresponding perpendicular diameter. To summarize these architectural traits and get an overall measure of wolf-willow patch characteristics, we first summed or averaged the measures at the plot-level, then used a principal component analysis (PCA) to ordinate the wolf-willow measurements: total canopy area, stem density, total basal area, average height and average canopy cover (**see****Supporting Information—Fig. S2**). Due to asexual reproduction by wolf-willow, stem density can be high both in very young and in mature stands. By using architectural measures, we hope to have a more sensitive analysis than simple shrub presence or stem density, examining how the density and size of wolf-willow within a stand influences the understory community. We termed the axis scores on the primary axis as ‘wolf-willow score.’ The score was positively associated with all traits, so a higher score generally corresponds to more wolf-willow. It should be noted that these traits likely have correlations to stand age (total basal area, average height) and stand developmental stage (total canopy cover, stem density). We did not quantify these relationships though, and as such will avoid making inferences linking score to stand age or developmental stage.

### Understory community composition and standing biomass

To investigate how wolf-willow and grassland plots differ in biodiversity and standing biomass, we sampled at the point scale recording species presence. All vegetation and litter sampling occurred at peak biomass (13–16 August). We collected peak above-ground herbaceous biomass via clipping, collected below-ground biomass using root cores (20 cm deep × 5 cm diameter) and collected litter via raking. Due to ongoing experiments at the site, we did not harvest wolf-willow above-ground biomass. Above-ground biomass and litter were dried at 60 °C for at least 48 h, then weighed. Below-ground biomass was field sieved and washed over a 2 mm sieve, dried and weighed. We were unable to reliably differentiate wolf-willow roots from other species, and for consistency weighed all roots occurring within a core, regardless of identity. As we were interested primarily in the root mass, any bulbs found were separated and weighed separately. During this process, we observed nodulation of wolf-willow roots, however, this was not quantified.

### Understory resource availability

To determine the effect of wolf-willow on soil resource availability, we measured soil moisture at each of the 25 points in each plot (13 August) using a Theta Probe (Delta-T Devices, Cambridge, UK). Though single measurements are unreliable, this serves as a coarse look at differences in soil moisture, rather than gathering detailed hydrological data. Due to cost considerations, we used a different sampling design to measure soil chemistry parameters, measuring these parameters at the quadrant scale. We took four soil cores (15 cm deep × 2 cm diameter) per quadrant (Fig. 1). Cores were then pooled by quadrant and sieved with a 2 mm sieve to discard any roots, creating four samples per plot. We used these samples to measure nitrogen and pH. Mineral nitrogen was extracted using 2.0 M KCl (Saha *et al.* 2018) and then measured by flow injection analysis (Lachat QuickChem QC8500 FIA Automated Ion Analyzer, Loveland, CO, USA) and pH was measured using a pH probe (ThermoFisher Scientific, Waltham, MA, USA). Like soil moisture, one-time sampling of nitrogen does not capture the long-term dynamics of soil chemistry. However, a one-time sampling can still provide insights into potential differences in inorganic N availability under similar environmental conditions.

To determine the effects of wolf-willow on light availability, we used hemispherical photography to measure the canopy cover at the 25 points within each wolf-willow plot (Fig. 1). We used a smartphone camera equipped with an Aukey 198° fisheye lens mounted on a selfie stick (Bianchi *et al.* 2017). Images were taken from ground level, post-vegetation clipping. Images were processed in ImageJ (Schneider *et al.* 2012) and we extracted the gap fraction using CIMES-FISHEYE (Gonsamo *et al.* 2011). To make the gap fraction measurements more intuitive we converted it to canopy closure (1 – Gap Fraction). We assumed a fixed height for canopies, based on the average height of shrub canopies within our study (1.08 m). We limited the search angle of the image analysis to 2.5 degrees (Θ = tan^-1^[point radius ÷ canopy height]), creating a search window the same size as our understory sampling points (5 cm radius).

### Statistical analysis

To quantify the spatial distribution of wolf-willow stems, we used a pooling procedure with Ripley’s L-function (Besag 1977; Baddely *et al.* 2015). We compared the observed stem distribution to a null model of random distribution, allowing us to identify scales at which stems were either clustered or overdispersed more than expected by chance.

To quantify the differences between wolf-willow and grassland plots in aspects of fertility and biomass stocks we used mixed-effects models comparing wolf-willow and grassland plots with block as a random effect. In eight separate models, we fit either above-ground biomass, below-ground biomass, litter, soil moisture, nitrogen (NO_3_, NH_4_, total mineral nitrogen) or pH as response variables. To assess the response of vascular plant diversity to wolf-willow presence, we used mixed-effects models with either richness or evenness (Pielou’s evenness index) as response variables, wolf-willow presence as a fixed effect and block as a random effect. The effects of wolf-willow presence on plant community composition were tested using a permutational multivariate analysis of variance (PERMANOVA) with 999 permutations. Wolf-willow presence was the predictor variable, and we incorporated the block design by constraining randomization to within a block. Before the ordination we used a Hellinger transformation on our species matrix to down-weight the importance of rare species (Legendre and Gallagher 2001).

To investigate how wolf-willow architecture is associated with our fertility, biomass and biodiversity parameters, we used 10 linear regressions, regressing each of above-ground biomass, below-ground biomass, litter, soil moisture, NO_3_, NH_4_, total mineral nitrogen, pH, average alpha diversity (richness per point within plot) and beta diversity (between points within plot) against wolf-willow score. Beta diversity was calculated using the *betadisper* function in R package vegan. This method is based on Anderson *et al.* (2006) and uses Euclidean distance to quantify the multivariate dispersion of plot plant community composition compared with the spatial median of all wolf-willow plots. Blocks were not specified in the model, as each block contains one wolf-willow plot.

To investigate the potential of wolf-willow to serve as an assembly filter we first used a constrained ordination, testing if plant composition was better explained by an environmental factor than by our community types. We used stepwise model selection to identify which of soil moisture, NO_3_, *NH*_*4*_, total mineral nitrogen, pH and community type best explained the data. Soil moisture and community type emerged as the top variables. We then used redundancy analysis and PERMANOVA with soil moisture as the predictor variable to determine how well community composition was explained by soil moisture.

To get at finer scale in changes in the understory community, we investigated single species’ responses to wolf-willow presence. We used generalized linear mixed-effects models testing the response of species abundance to a community type fixed effect. For this analysis, we defined species abundance as fraction of total sampling points with observed species’ presence. Block was inputted as a random effect. We tested all species, as our interest was primarily in seeing if wolf-willow and grassland communities exhibit differences in assembly, rather than making conclusions about specific species’ responses to either of the community types.

All analyses were done in R 3.5.3 (R Core Team 2019), using packages *spatstat* (Baddely *et al.* 2015), *lme4* (Bates *et al.* 2015) and *vegan* (Oksanen *et al.* 2019).

## Results

### Wolf-willow structural and spatial characteristics

Within our shrub plots, wolf-willow densities ranged from 1.78 to 5.22 stems/m^2^ with an average density of 2.91 ± 0.40 stems/m^2^ (mean, SE). Average canopy closure was 13.3 ± 1.19 %, with a range of 4.3–24.7 %. Stems were significantly clustered spatially at small scales (4–30 cm) but were randomly distributed at larger scales (31–80 cm) (**see**Supporting Information**—Fig. S1**). No wolf-willow individuals were detected in the grassland plots.

### Patterns of wolf-willow presence

**Figure 2.**
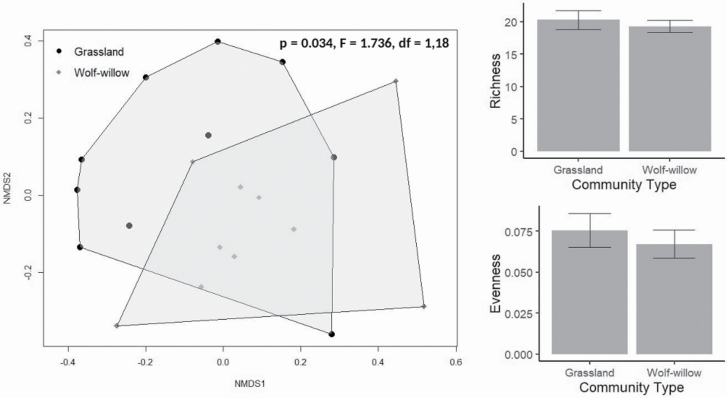
The effects wolf-willow (*Elaeagnus commutata*) presence on community composition in the Aspen Parkland. On the left, an ordination of the compositional data, with the results of a PERMANOVA in the top right of the plot. On the right, two bar plots showing the differences in richness (top), and evenness (bottom) between the two community types, neither richness nor evenness were significantly different (df = 9 for both, *t* = -0.653, *P* = 0.531 and *t* = -1.7098, *P* = 0.122, respectively). Error bars are 1 standard error.

**Figure 3.**
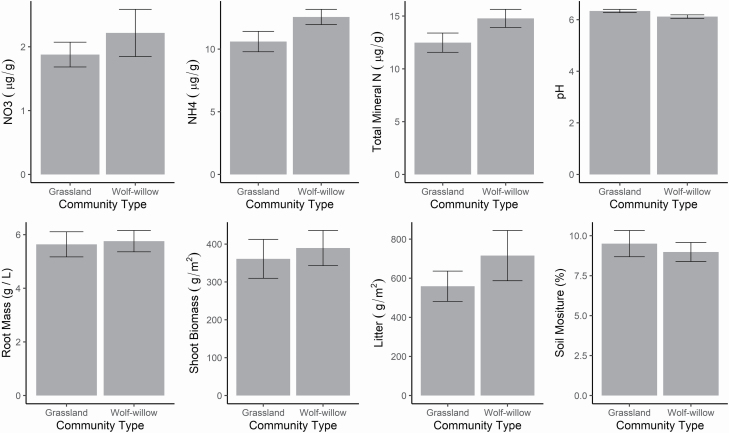
Differences between grassland and wolf-willow (*Elaeagnus commutata*) dominated communities in fertility and biomass stocks. Results of mixed effects models on these data are presented in Table 2. Error bars are 1 standard error.

Wolf-willow had few associations with core community metrics. Despite an average canopy cover of 13 %, we found no difference between wolf-willow dominated and grassland community types for species richness, biomass, soil nutrients or soil moisture (Table 2; Fig. 3). In contrast, wolf-willow stands were associated with greater litter biomass and lower soil pH (Table 2; Fig. 3). Neither species richness nor evenness differed between wolf-willow and grassland community types (Table 2; Fig. 2); however, the NMDS and PERMANOVA showed a significant divergence in composition (Fig. 2).

**Table 2.** Results of linear mixed effects models on the effect of wolf-willow presence on factors of the understory community. All tests had 1,8 degrees of freedom, and all values are rounded to three decimal places.

Measurement	*F*	*P*
Soil pH	5.457	0.044
Soil ammonium (NH^+^_4_)	3.557	0.093
Soil total mineral nitrogen	3.265	0.104
Soil nitrate (NO^−^_3_)	0.978	0.348
Soil moisture	0.476	0.508
Litter mass	5.541	0.043
Above-ground biomass	2.969	0.119
Below-ground biomass	0.068	0.801
Richness	0.425	0.531
Evenness	2.924	0.122

### Effects of wolf-willow architecture

**Figure 4.**
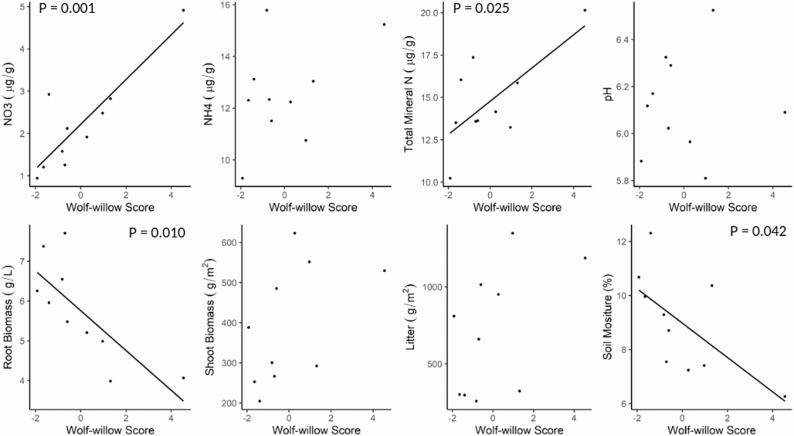
Linear models of how wolf-willow (*Elaeagnus commutata*) score relates to biomass stocks and fertility components of understory communities. Wolf-willow score is derived from Axis 1 on a PCA of wolf-willow structural traits. A line of best fit indicates a significant relationship between the two variables.

The lack of overall effects from the binary presence/absence of wolf-willow masks variation observed within wolf-willow stands. The PCA generated a primary axis that explained 73.8 % of variation and was positively associated with all inputted measures (**see****Supporting Information—Fig. S2**). As our wolf-willow score increased, there was significant positive association with NO_3_ (F = 5, df = 1,8, *P* = 0.001) and total mineral nitrogen (F = 7.513, df= 1,8, *P* = 0.025) and significant negative associations with root biomass (F = 2.924, df = 1,8, *P* = 0.0102) and soil moisture (F = 5.813, df = 1,8, *P* = 0.042) (Fig. 4). The total mineral nitrogen association is likely driven by the NO_3_ association, as NH_4_ had weak evidence of association (F = 2.039, df = 1,8, *P* = 0.191). Wolf-willow had no effect on average alpha or beta diversity (F = 2.786, F = 0.664, df = 1,8 both, *P* = 0.134, 0.439, respectively) or plot-level species richness (F = 0.893, df = 1,8, *P* = 0.893), though there was a trend towards reduced species evenness as the wolf-willow score increased (F = 3.980, df = 1,8, *P* = 0.081).

In the constrained ordination, we did not find evidence of an environmental assembly filter driving our observed plant composition. The primary axis of soil moisture explained 8.8 % of the variation in composition. The PERMANOVA showed that soil moisture was not as strong a predictor of composition as community type (*P* = 0.093, F = 0.187, df = 1,18, compared with *P* = 0.034 for community type).

**Figure 5.**
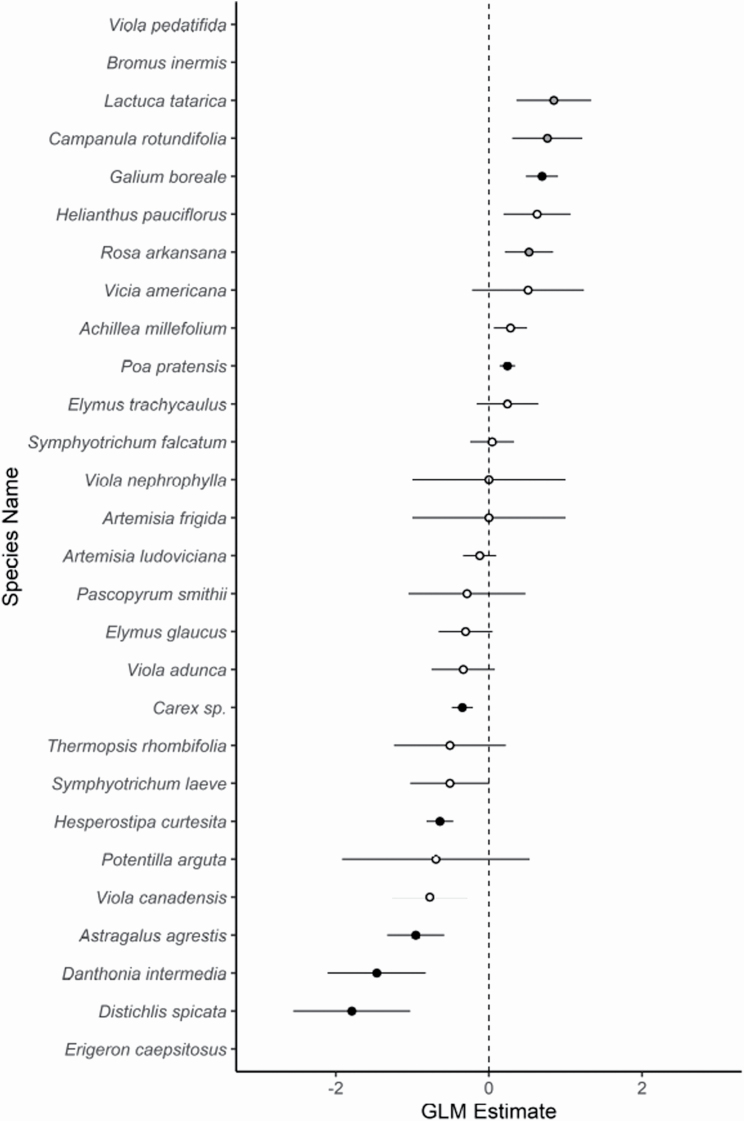
Response of species counts to wolf-willow presence. Error bars represent 1 standard error. Point fill corresponds to significance. White is *P* ≥ 0.1, grey is 0.1 > *P* ≥ 0.05 and black is *P* < 0.05. *Erigeron caespitosus, Bromus inermis* and *Viola pedatifida* all had extreme values, and are not shown in the plot area. Exact values for all species can be found in Table 3.

Beneath the breadth of mostly null results at the community levels, we did find evidence that wolf-willow influences certain species within the grassland. Among species, there is a response gradient to wolf-willow presence, with species’ responses ranging from significantly negative to significant positively (Table 3; Fig. 5).

**Table 3.** Species’ count responses to wolf-willow presence. Using a GLM with a Poisson distribution, differences in species counts between grassland and wolf-willow communities were analysed. A negative estimate indicates bias towards grassland, while a positive estimate indicates bias towards wolf-willow. Species with singular fitting models or model errors were excluded.

Species Name	Estimate	SE	Z	*P*
*Distichlis spicata*	-1.7918	0.7638	-2.346	0.019
*Danthonia intermedia*	-1.4663	0.6404	-2.29	0.022
*Astragalus agrestis*	-0.9555	0.3721	-2.568	0.0102
*Hesperostipa curtesita*	-0.6408	0.1745	-3.671	0.000241
*Carex sp.*	-0.3477	0.138	-2.52	0.0117
*Poa pratensis*	0.24 116	0.10 068	2.395	0.0166
*Galium boreale*	0.6931	0.2094	3.309	0.000935
*Erigeron caepsitosus*	-31.28	6 196 000	0	1
*Viola canadensis*	-0.77 317	0.49 354	-1.567	0.117
*Potentilla arguta*	-0.6931	1.2247	-0.566	0.5714
*Symphyotrichum laeve*	-0.5108	0.5164	-0.989	0.323
*Thermopsis rhombifolia*	-0.5108	0.7303	-0.699	0.484
*Viola adunca*	-0.3365	0.414	-0.813	0.416
*Elymus glaucus*	-0.3054	0.3522	-0.867	0.386
*Pascopyrum smithii*	-0.2877	0.7638	-0.377	0.706
*Artemisia ludoviciana*	-0.1206	0.2163	-0.558	0.577
*Artemisia frigida*	-9.822E-07	1	0	1
*Viola nephrophylla*	4.476E-05	1	0	1
*Symphyotrichum falcatum*	0.04082	0.28 544	0.143	0.886
*Elymus trachycaulus*	0.24 116	0.40 291	0.599	0.549
*Achillea millefolium*	0.2809	0.2172	1.294	0.196
*Vicia americana*	0.5108	0.7301	0.7	0.4841
*Rosa arkansana*	0.5232	0.3155	1.659	0.0972
*Helianthus pauciflorus*	0.6286	0.4378	1.436	0.151
*Campanula rotundifolia*	0.7621	0.4577	1.665	0.0959
*Lactuca tatarica*	0.8473	0.4879	1.737	0.0825
*Bromus inermis*	31.13	883.37	0.035	0.972
*Viola pedatifida*	39.78	5792.62	0.007	0.995

## Discussion

Though wolf-willow is a dominant plant in the Aspen Parkland, our results show that its presence alone is associated with relatively minor changes in community composition, understory biomass and soil resource availability. These results run counter to many shrub encroachment studies, where the establishment of shrubs can have strong negative effects on the species richness and productivity of the herbaceous species in a system (Briggs *et al.* 2002, Price and Morgan 2008, Castro and Freitas 2009, López-Díaz *et al.* 2015). These negative effects can arise due to increased litter deposition (Hilger and Lamb 2017; Zhang *et al.* 2019). While we saw increased litter in our shrub understories, we did not detect an effect on richness or standing biomass. Though we observed equal above-ground biomass between the two community types, our biomass measures for wolf-willow stands excluded wolf-willow biomass. Had we measured this we likely would have found shrub stands to have higher above-ground biomass than grasslands. Our effect sizes are likely driven by the architectural traits of wolf-willow stands, where the low stem density, sparse canopy and clumped arrangement constrain the strength of effect that wolf-willow has on the communities where it occurs (Bork and Burkinshaw 2009; Sabo *et al.* 2009).

Based on the number of significant relationships, our ‘wolf-willow score’ analyses seem to be more sensitive than our presence/absence comparisons. The associations between wolf-willow and soil nitrogen and pH are likely driven by interactions between nitrogen fixation and litter deposition. The observed nitrogen enrichment may have arisen directly through fixation (Paul *et al.* 1971), or through deposition and decomposition of leaf litter (Siemann and Rogers 2003). Acidification in wolf-willow stands could be driven indirectly by fixation-derived ammonium ions. Plant use of ammonium can acidify the rhizosphere (von Wirén *et al.* 2000) while nitrification of ammonium releases H^+^ ions into the soil (Cole 1995). Both acidification and nitrogen enrichment can have impacts on the understory plant community, altering the bioavailability of NO^−^_3_/NO^−^_2_ (Kemmitt *et al.* 2005), as well as the nodulation of legume roots (Coventry *et al.* 1985). Allelopathy is one plausible explanation of the observed changes in soil chemistry. Other studies on allelopathy have observed changes in soil pH resulting from allelochemical inputs (Morris *et al.* 2012; Uddin *et al.* 2020). A congener has shown some evidence of allelopathic effects (Orr *et al.* 2005), as well as altering rates of nitrification in soil (Lunares *et al.* 1993), but more detailed work is needed on wolf-willow to investigate this possibility. Our soil chemistry measurements lack a temporal dimension and adding this data may help to solidify our understanding of wolf-willow presence on soil, as well as how these conditions develop.

There are two possible explanations to the observed negative association between wolf-willow score and root biomass. Wolf-willow could be reducing overall root biomass in the system, and thus changing root:shoot ratios of understory plants. However, because we did not sample wolf-willow shoot biomass, it is difficult to say conclusively how root:shoot ratios may differ between grasslands and shrub stands. Other studies have found understories of woody plants to have lower root:shoot ratios than grasslands (Wilson 1993; Grouzis and Akpo 1997; Pinno and Wilson 2011). Examples of studies finding declines in overall root mass associated with increasing shrub presence are difficult to find (but see Yusuf *et al.* 2015), so at this time we lack a clear mechanism to support this hypothesis. Allelopathy (mentioned previously) may be responsible for declines in root mass (Orr *et al.* 2005), but without clear evidence of wolf-willow allelopathy this is merely speculation. The second possibility explaining observed root mass declines is that wolf-willow roots deeper than many grassland species, causing a deeper allocation of root biomass (thus out of range of our cores). This could be evidence of soil niche partitioning (Kulmatiski and Beard 2013), though other studies on niche partitioning of grass and shrub roots typically see grass rooting become shallower rather than deeper in response to shrub presence (Peláez *et al.* 1994, Nippert and Holdo 2015). In the absence of root:shoot allocational shifts, this explanation would also logically predict lower herbaceous above-ground biomass, as deeper allocation explaining lower biomass assumes a zero-sum scenario of root biomass. The fact that we do not see reduced shoot biomass makes it unlikely for this to be the sole explanation, though the true process might involve components of both explanations presented here. Grasslands are important carbon sinks (Bengtsson *et al.* 2019), and the potential for wolf-willow presence to reduce root mass in grasslands may be detrimental to the provisioning of this service, particularly at high shrub densities.

Plot-level richness seems to be too coarse a measure to detect wolf-willow effects on understory community composition. Using more sensitive analyses, we can see evidence that wolf-willow is affecting the community composition of the system. Our ordination plot shows minor divergence, supported by a marginally significant PERMANOVA. Evidence of wolf-willow fine-tuning the understory composition can further be seen by the evenness response. The negative association between wolf-willow score and evenness suggests that dominance increases in wolf-willow understories. A prime beneficiary of this effect is *Poa pratensis*, a dominant grass in the Aspen Parkland (Lamb 2008), which showed a significant positive association to wolf-willow presence. However, not all dominant species increase under wolf-willow. *Hesperostipa curtesita* is another dominant grass in this system (Lamb 2008), yet showed a significant negative association with wolf-willow. While many studies report richness effects associated with shrub presence (Anthelme *et al.* 2007; Price and Morgan 2008; Ratajczak *et al.* 2012; Madsen *et al.* 2020), our study shows that important community changes can occur even in the absence of a richness effect. Future studies should consider incorporating architectural or abundance measures (like our wolf-willow score) as well as looking at other components of biodiversity.

Though wolf-willow presence dramatically alters the visual landscape of grasslands in the Aspen Parkland, the realized changes in the grassland community we observed are more subtle, supporting a nuanced view of community dominance (Grieg-Smith 1986; Frieswyk *et al.* 2007). With evidence of changes in soil chemistry, soil moisture and below-ground biomass, wolf-willow seems to be associated with important below-ground processes; however, to understand both the mechanisms and outcomes, more detailed studies are needed. The results of our study provide baseline information on the functionality of wolf-willow dominated areas in the Aspen Parkland. The results of our architectural approach suggest that architecture may be a currently understudied driver of shrubland dynamics. By incorporating architectural traits and spatial arrangement, we might more accurately compare shrubland dynamics across species and ecosystems, aiding in the development of a more generalized understanding of shrubland patterns and processes.

## Supporting Information

The following supporting information is available in the online version of this article—

**Figure S1.** Summarizes wolf-willow physical and spatial characteristics, including the plot of the L-Function.

**Figure S2.** The results of the PCA on wolf-willow architectural measures and how they relate to wolf-willow score (the primary PCA axis).

plab011_suppl_Supplementary_Figures

## Data Availability

All code and data used in this study are available at Peetoom Heida, Isaac; Brown, Charlotte; Dettlaff, Margarete A.; Oppon, Kenneth J.; and Cahill, James F. (2021): Presence of a dominant native shrub is associated with minor shifts in the function and composition of grassland communities in a northern savannah. Figshare. Dataset. https://doi.org/10.6084/m9.figshare.13697590

## Notes

**Populations & Communities. Chief Editor:** Jean Burns
