# Supplementary material for: Presence of a dominant native shrub is associated with minor shifts in the function and composition of grassland communities in a northern savannah
Source: AoB Plants. 2021 Feb 23;13(2):plab011. doi: 10.1093/aobpla/plab011 (PMC8050699; doi:10.1093/aobpla/plab011)
Supplement: plab011_suppl_Supplementary_Figures [file plab011_suppl_supplementary_figures.pdf]

Supplemental Figures

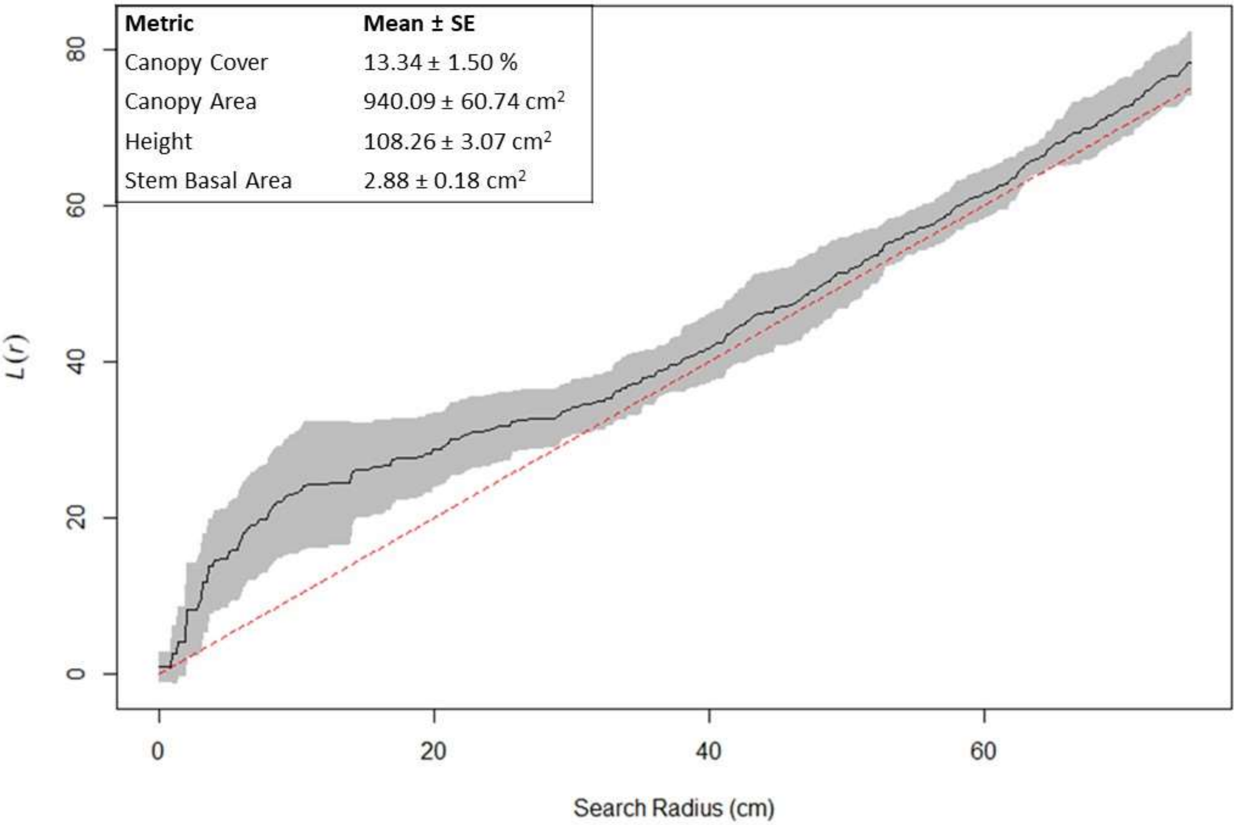

Figure S1. Physical characteristics of wolf-willow (*Elaeagnus commutata*) and patterns of stem aggregation. 262 stems were mapped across 10 plots, and measurements of each stem were taken. The plot shows the results of an L-function on our pooled maps. The red dashed line represents the null hypothesis of random aggregation. In black, the observed pattern of wolf-willow stems, bounded by a simulation envelope. Deviations above the null line indicate clumping, while deviations below indicate overdispersion.

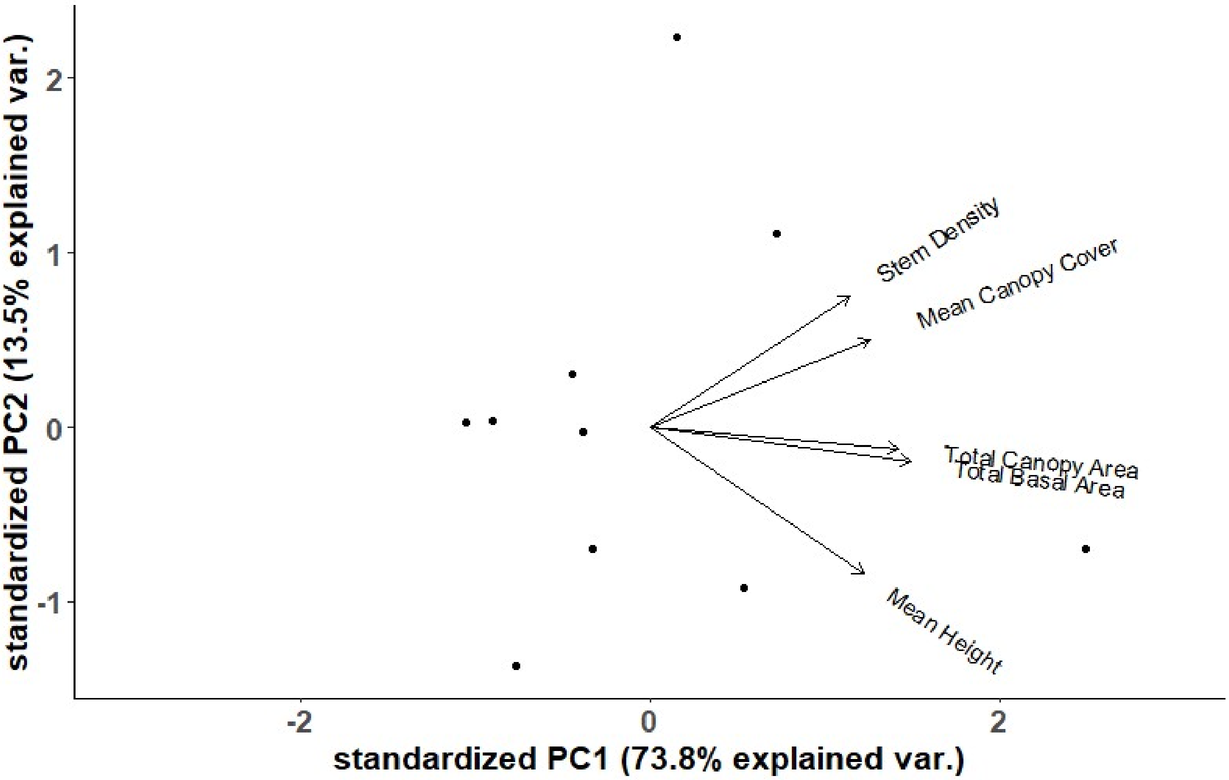

Figure S2. Principal Components Analysis of wolf-willow stand architecture. Ten 3x3m plots had all wolf-willow stems measured, and canopy cover assessed at 25 points. After PCA, each plot's score on the primary axis was used as the "wolf-willow score" to give a summary value of the architecture of the plot. Vector arrows show the relationship of each measurement to the primary axis.
